# High-rate dark fermentative hydrogen production in a UASB reactor with granular activated carbon and *Clostridium pasteurianum* bioaugmentation

**DOI:** 10.3389/fmicb.2026.1890141

**Published:** 2026-08-04

**Authors:** Eunjin Kim, Gi-Beom Kim, Yerim Lee, Eunseo Cho, Sandhya Sompura, Jiho Jeon, Sang-Hyoun Kim, Ju-Hyeong Jung

**Affiliations:** 1 Department of Environmental Engineering, Kunsan National University, Gunsan, Republic of Korea; 2 Department of Biological and Agricultural Engineering and Texas A&M AgriLife Research Center, Texas A&M University, College Station, TX, United States; 3 Department of Civil and Environmental Engineering, Yonsei University, Seoul, Republic of Korea

**Keywords:** bioaugmentation, *Clostridium pasteurianum*, dark fermentation, granular activated carbon, microbial community analysis

## Abstract

The combined application of granular activated carbon (GAC) and *Clostridium pasteurianum* bioaugmentation was evaluated in an up-flow anaerobic sludge blanket (UASB) reactor for high-rate dark fermentative hydrogen production under progressively shortened hydraulic retention times (HRTs). Two parallel reactors were operated: a control reactor (UASB-C) and a modified reactor incorporating both GAC and repeated bioaugmentation (UASB-ACBA). Compared with UASB-C, UASB-ACBA achieved a 26% higher maximum hydrogen yield (1.94 moL H_2_/moL glucose consumed) and an approximately fourfold higher hydrogen production rate (83.08 L/L/d), while maintaining hydrogen production at an HRT of 1 h. Metabolite profiles suggested that UASB-ACBA maintained a more balanced lactate-associated fermentation pattern under shortened HRT conditions, whereas UASB-C showed lactate, acetate, and residual glucose accumulation under intensified loading. Microbial community analysis indicated the persistence of the ASV assigned to *C. pasteurianum* and the coexistence of hydrogen-producing Clostridium species and lactic acid-producing bacteria. Overall, GAC addition and *C. pasteurianum* bioaugmentation may support high-rate UASB dark fermentation.

## 1 Introduction

Fossil fuels remain the dominant global energy source and continue to contribute substantially to greenhouse gas emissions and global warming (Osman et al., 2023). Therefore, interest in sustainable and clean energy alternatives has increased, and biohydrogen has emerged as a promising option because it can be produced under mild conditions with relatively low greenhouse gas emissions (Eroglu and Melis, 2011; Lin et al., 2017; Noblecourt et al., 2018) . Among biological hydrogen production pathways, dark fermentation is particularly attractive because it does not require light, has relatively low energy requirements, and can utilize diverse organic wastes and biomass (Baeyens et al., 2020; Martínez-Fraile et al., 2024; Nikolaidis and Poullikkas, 2017). These characteristics make dark fermentation suitable for waste-to-energy applications by enabling hydrogen production from organic residues under relatively low-energy operating conditions.

An effective reactor design is essential for stable and efficient dark fermentation under high-throughput conditions, where shortened hydraulic retention times (HRTs) and elevated organic loading rates (OLRs) can impose substantial hydraulic and organic loading stress on hydrogen-producing microbial communities (Alexandropoulou et al., 2018; Carrillo-Reyes et al., 2014; Han et al., 2016; Jung et al., 2011; Show et al., 2011). The upflow anaerobic sludge blanket (UASB) reactor is suitable for dark fermentation because it retains high microbial biomass and enables stable operation at elevated OLRs (Zhao et al., 2008). However, high-throughput operation, typically achieved by reducing the HRT, can disturb hydrogen-producing pathway dominance and process stability (Moreno Dávila et al., 2020; Sivagurunathan et al., 2016). These challenges are mainly associated with microbial community restructuring, washout of key functional microorganisms, and limitations in interspecies electron transfer (Carrillo-Reyes et al., 2012; Chang and Lin, 2004). Therefore, strategies that improve microbial retention, strengthen hydrogen-producing activity, and support favorable microbial interactions are required under shortened HRT conditions.

Recent strategies have focused on improving microbial retention and steering microbial community functions under intensified fermentation conditions (Feng et al., 2024; Park et al., 2018). Conductive materials (CMs) have been reported to facilitate microbial attachment and electron transfer-associated interactions by providing conductive surfaces among microorganisms, thereby improving cooperative metabolism and hydrogen production (Cheng et al., 2020; Yang et al., 2024). Granular activated carbon (GAC) is particularly attractive because it is chemically stable, widely available, and provides a high-surface-area scaffold for microbial attachment and potential conductive surface-associated interactions (Mohd Jamaludin et al., 2023). Bioaugmentation is another complementary strategy, in which selected hydrogen-producing microorganisms are introduced to reinforce the functional capacity of native microbial communities and accelerate the establishment of hydrogen-producing dominance (Ács et al., 2015; Villanueva-Galindo and Moreno-Andrade, 2021). In particular, *Clostridium pasteurianum* is a representative dark-fermentative bacterium capable of converting organic substrates into hydrogen through acidogenic metabolism.

Although GAC addition and bioaugmentation have each been investigated to improve anaerobic fermentation performance, their combined application in dark-fermentative UASB reactors remains insufficiently understood (Kumar et al., 2016; Zinatizadeh et al., 2017). Under shortened HRT conditions, bioaugmented hydrogen-producing bacteria may be washed out or outcompeted by indigenous microorganisms, limiting their long-term contribution to hydrogen production (Menzel et al., 2023). In this context, GAC may provide attachment sites for microbial retention and conductive surfaces that could support microbial interactions, thereby helping maintain the persistence and activity of the introduced hydrogen-producing strain (Quintelas et al., 2009). Therefore, the simultaneous application of GAC and *C. pasteurianum* bioaugmentation may provide complementary benefits by combining GAC-supported biomass retention with the targeted introduction of a hydrogen-producing bacterium. However, whether this combined strategy can stabilize and intensify hydrogen production under progressively decreasing HRT conditions remains unclear. To address this research gap, this study evaluated whether the combined application of GAC and *C. pasteurianum* bioaugmentation could improve hydrogen production and process stability in a dark-fermentative UASB reactor under progressively shortened HRT conditions.

## 2 Materials and methods

### 2.1 Preparation of inoculum, substrate, and additives

#### 2.1.1 Inoculum and feedstock preparation

The startup inoculum was anaerobic digester sludge collected from an anaerobic digester at a municipal wastewater treatment plant in Bucheon, Republic of Korea. The inoculum was not pre-granulated sludge. The properties of the anaerobic sludge were as follows: total solids (TS): 40.01 ± 0.15 g/L, volatile solids (VS): 21.13 ± 0.04 g/L, total suspended solids (TSS): 38.15 ± 0.07 g/L, volatile suspended solids (VSS): 20.64 ± 0.48 g/L and pH: 7.65 ± 0.12. The anaerobic sludge was pretreated at 90 °C for 1 h to deactivate the methanogens. The feedstock composition was as follows: 20 g/L of glucose, 3 g/L of NH_4_HCO_3_, 125 mg/L of KH_2_PO_4_, 100 mg/L of MgCl_2_⋅6H_2_O, 25 mg/L of FeSO_4_⋅7H_2_O, 15 mg/L of MnSO_4_⋅6H_2_O, 5 mg/L of CuSO_4_⋅5H_2_O, and 1 mg/L of CoCl_2_⋅5H_2_O (Park et al., 2017). To maintain pH at 5.5–6.0, NaHCO_3_ was dosed at 4.0–9 g/L in response to the measured reactor pH.

#### 2.1.2 Bioaugmentation and conductive material

*Clostridium pasteurianum* was used for bioaugmentation. *C. pasteurianum* was selected because it is a representative dark-fermentative hydrogen-producing bacterium with high hydrogen-producing capacity from glucose and proven applicability in continuous biohydrogen systems (Hung et al., 2007). The strain was obtained from the Korean Collection for Type Cultures (KCTC; Daejeon, Republic of Korea) and cultivated at 37°C and 150 rpm in Reinforced Clostridial Broth (RCB; MB-R1602, MBcell, Republic of Korea) prepared at 37.5 g/L and autoclaved (121°C, 15 min). The cells were harvested at an optical density (OD600) of 4.0 and used as the bioaugmentation inoculum. The inoculum dose was controlled based on OD600 and culture volume (Okonkwo et al., 2020; Sim et al., 2024). Commercial granular activated carbon (GAC, CJG type, 4 × 8 mesh) was used as the electrically conductive carbon material. GAC was obtained from Cheongjeong Chemical (Gunsan, Republic of Korea) and sieved through a 2 mm mesh before use. The basic physicochemical properties of the GAC were as follows: specific surface area of approximately 1,000–1,600 m^2^/g, iodine adsorption capacity of 950–1,150 mg/g, bulk density of 0.45–0.52 g/mL, hardness of ≥ 95%, moisture content of ≤ 5%, specific gravity of 1.8–3.51 and particle size of < 2 mm.

### 2.2 UASB reactor: experimental setup and operation

**FIGURE 1:**
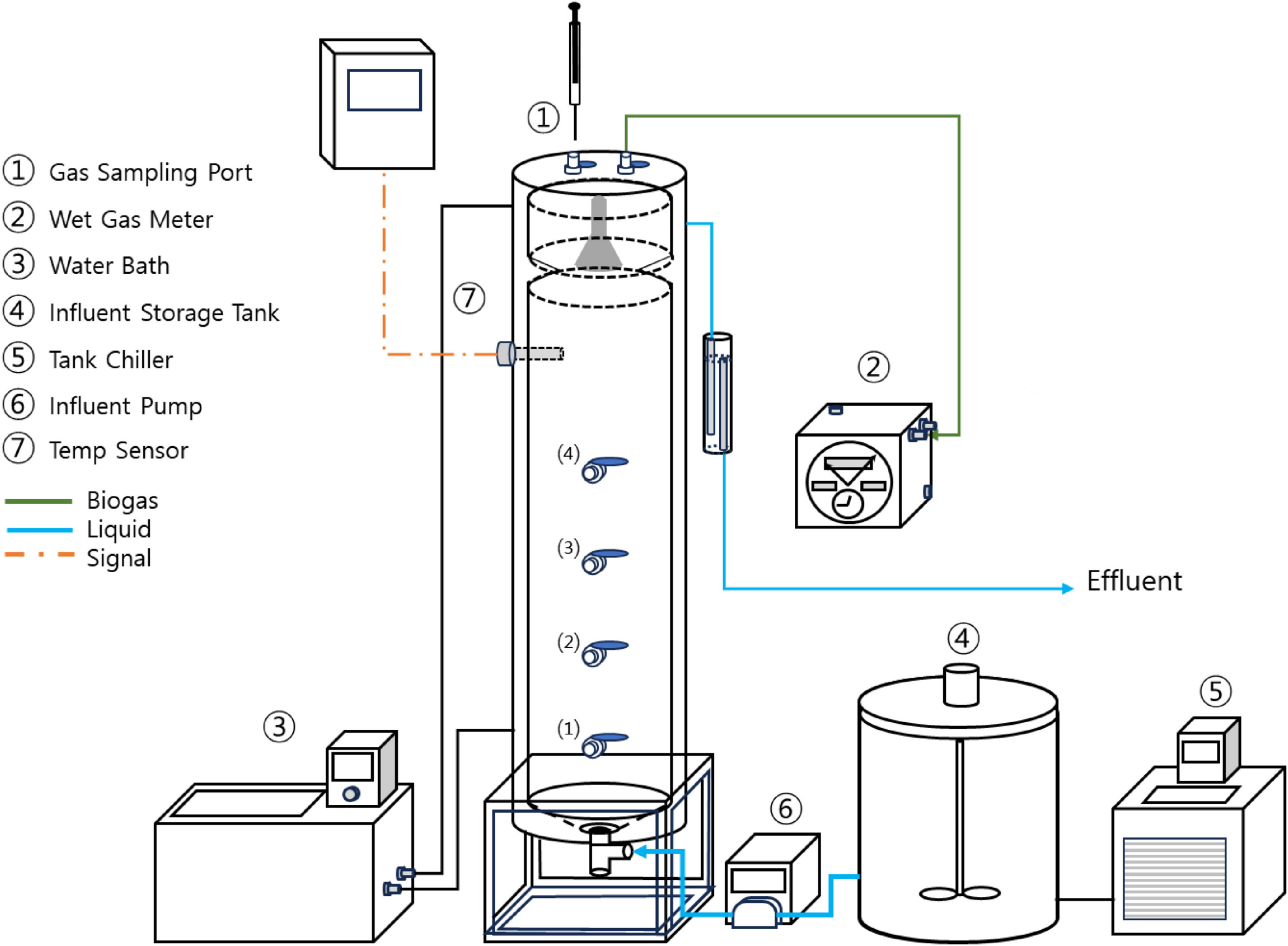
Schematic diagram of UASB system.

Up-flow anaerobic sludge blanket (UASB) reactors were operated in an anaerobic mesophilic (35 ± 1°C) condition (Figure 1). The total volume and working volume were 5.5 and 5 L, respectively. Startup inoculation was performed by mixing 1 L of pretreated anaerobic sludge with 4 L of feedstock in both the UASB-C (control) and UASB-ACBA (activated carbon + bioaugmentation) reactors. Based on the VSS concentration of the pretreated anaerobic sludge, the initial VSS concentration in each UASB reactor after inoculation was approximately 4.13 g VSS/L. In the UASB-ACBA reactor, GAC was added at 5% (w/v) of the working volume during startup. The continuous operation was divided into five periods based on the applied hydraulic retention time (HRT): 12 h (Period I), 6 h (Period II), 3 h (Period III), 2 h (Period IV), and 1 h (Period V, UASB-ACBA only). Bioaugmentation with *C. pasteurianum* was first conducted when the operation was shifted from Period I to Period II and was subsequently repeated whenever the HRT was shortened. At each bioaugmentation event, *C. pasteurianum* culture at OD600 = 4.0 equivalent to 20% of the reactor working volume was introduced into the reactor. The culture was directly added without prior withdrawal of reactor liquid. The resulting excess volume was immediately discharged through the overflow outlet, thereby restoring the reactor working volume to 5L. This dose was selected to ensure sufficient introduction of the target hydrogen-producing strain and to reduce the risk of immediate loss of the introduced population under shortened HRT conditions. Bioaugmentation was not applied during Period I because this period was used as an initial adaptation and baseline stabilization phase for the heat-pretreated anaerobic sludge before introducing the selected strain. The UASB systems were initially operated in batch mode for 36 h after inoculation and then transitioned to continuous mode when the reactor pH reached 5.5. In the continuous mode, the feedstock was continuously fed into the reactors at the bottom inlet using a peristaltic pump in accordance with the HRT, and the effluent was discharged as overflow from the top. The influent flow rate was not adjusted during the bioaugmentation procedure. Data collected immediately after each bioaugmentation event were excluded from the pseudo-steady-state performance calculations to avoid transient hydraulic and dilution effects associated with culture addition. The control reactor was not further operated at 1h HRT because HPR and reactor-retained VSS declined at 2h HRT, indicating deterioration of reactor performance under the intensified hydraulic loading. Therefore, only UASB-ACBA was further operated at 1h HRT to evaluate hydrogen production under more intensified hydraulic loading. The HRT was changed to the next level after pseudo-steady-state performance was maintained for more than 4 consecutive days, during which hydrogen production varied within ± 10%. The volumetric organic loading rate (OLR) was calculated based on the influent glucose COD concentration, and the specific loading rate (SLR) was calculated by dividing the OLR by the average reactor-retained VSS concentration during the pseudo-steady-state phase of each period. The operational conditions, including the duration of each operational period and the number of days used for pseudo-steady-state averaging, are summarized in Table 1. The biogas volume was quantified using a wet gas meter (W-NK-0.5BE, Shinagawa Corporation, Tokyo, Japan). The biogas was normalized to standard conditions of 1 atm and 0°C.

**TABLE 1:** Average continuous hydrogen production performance and volatile suspended solids (VSS) for each period.

Reactor	Parameters	Period I (12 h HRT)	Period II (6 h HRT)	Period III (3 h HRT)	Period IV (2 h HRT)	Period V (1 h HRT)
UASB-ACBA	OLR (g COD/L/d)	42.68	85.36	170.72	256.08	512.16
	SLR (g COD/g VSS/d)	33.09	35.57	37.85	28.26	24.19
	Hydrogen production rate (L/L/d)	7.08 ± 0.88	16.78 ± 0.62	35.80 ± 1.32	56.96 ± 1.33	83.08 ± 2.34
	Hydrogen yield (moL H_2_/moL glucose_added_)	1.42 ± 0.18	1.69 ± 0.06	1.80 ± 0.07	1.91 ± 0.04	1.39 ± 0.04
	Hydrogen yield (moL H_2_/moL glucose_consumed_)	1.46 ± 0.18	1.79 ± 0.05	1.83 ± 0.08	1.94 ± 0.03	1.49 ± 0.04
	Volatile suspended solids (g VSS/L)	1.29 ± 0.02	2.40 ± 0.14	4.51 ± 0.18	9.06 ± 0.17	21.17 ± 2.97
	Operation period (d)	6	18	30	20	27
	Pseudo-steady-state period (d)	4	8	12	8	10
UASB-C	OLR (g COD/L/d)	42.68	85.36	170.72	256.08	-
	SLR (g COD/g VSS/d)	29.43	34.42	42.15	83.41	−
	Hydrogen production rate (L/L/d)	7.28 ± 1.33	12.49 ± 1.26	20.46 ± 1.84	17.52 ± 1.93	-
	Hydrogen yield (moL H_2_/moL glucose_added_)	1.46 ± 0.27	1.25 ± 0.13	1.03 ± 0.09	0.59 ± 0.06	−
	Hydrogen yield (moL H_2_/moL glucose_consumed_)	1.54 ± 0.30	1.39 ± 0.15	1.29 ± 0.17	0.79 ± 0.09	−
	Volatile suspended solids (g VSS/L)	1.45 ± 0.06	2.48 ± 0.19	4.05 ± 0.24	3.07 ± 0.01	−
	Operation period (d)	8	23	24	4	
	Pseudo-steady-state period (d)	4	8	12	4	

OLR was calculated from the influent glucose COD concentration of 21.34 g COD/L and HRT. SLR was calculated by dividing OLR by the average reactor-retained VSS concentration during the pseudo-steady-state period.

The continuous reactor experiment was conducted using one reactor for each condition. Average values and standard deviations were calculated from daily measurements obtained during the pseudo-steady-state period of each HRT condition. No intentional sludge wasting was performed; therefore, the solids retention time was not externally controlled.

### 2.3 Hydrogen producing activity (HPA)

A series of batch hydrogen-producing activity (HPA) tests were performed in triplicate using biomass collected under each reactor operating condition, and the results are presented as mean ± standard deviation. Granule biomass samples were collected from each reactor at the end of the pseudo-steady-state phase of each HRT condition and immediately used as inocula for the HPA tests. Period I was excluded from the HPA tests because it was regarded as the initial startup and adaptation phase before bioaugmentation and before exposure to shortened HRT conditions. Granules collected from the reactors were inoculated into 160 mL serum bottles at 10% (v/v), resulting in a working volume of 100 mL. The VSS concentration was not adjusted to a fixed value but was determined from the granule biomass collected from each reactor. This approach was adopted because the objective of the HPA test was to evaluate the hydrogen-producing activity of the reactor-retained biomass under each operating condition. The substrate composition was identical to that of the continuous-mode feedstock, and the initial pH was adjusted to 8.0 using NaHCO_3_. The substrate-to-inoculum (S/I) ratios, calculated as the initial glucose COD to inoculum VSS, are summarized in Table 2. Because the inoculum VSS concentration differed among the operating periods, the S/I ratio also varied accordingly. All bottles were purged with nitrogen for 3min and immediately crimp-sealed to maintain strict anaerobic conditions. Hydrogen fermentation was conducted in a shaking incubator at 37 ± 0.3°C and 150 rpm until gas production ceased. The cumulative hydrogen production over time was fitted using Equation 1


$$H=P\times \text{exp}[-\text{exp}\left\{\frac{R_{H}}{P}\times \left(\lambda -t\right)\times e+1\right\}$$ (1)

**TABLE 2:** Hydrogen-producing activity under each operating condition.

Reactor	Period	HY (moL H_2_/moL glucose_added_)	HPR (mL H_2_/h)	S/I ratio (g COD/g VSS)	Lag phase (h)	*R* ^2^	Biomass specific HY (mL H_2_/g VSS)
UASB-ACBA	Period II	1.39 ± 0.11	9.15 ± 0.26	8.92	39.0	0.999	32.20
	Period III	1.09 ± 0.05	11.85 ± 0.43	4.75	59.0	0.999	18.32
	Period IV	1.12 ± 0.05	9.78 ± 1.01	2.36	52.1	0.993	9.72
	Period V	1.47 ± 0.05	11.19 ± 0.88	1.01	26.9	0.994	5.61
UASB-C	Period II	1.42 ± 0.07	6.87 ± 0.32	8.63	93.8	0.996	22.99
	Period III	1.38 ± 0.04	7.75 ± 0.74	6.28	63.3	0.990	29.19
	Period IV	1.16 ± 0.06	10.01 ± 0.24	6.97	81.5	0.999	33.25

Values are presented as mean ± standard deviation of triplicate batch tests. No statistical hypothesis testing was performed; therefore, differences between reactors were interpreted descriptively.

where *H*, *P*, *R*_*H*_, λ, and *t* are the cumulative hydrogen production (CHP; mL), maximum hydrogen production (mL), HPR (mL/h), lag phase (h), and time (h), respectively.

### 2.4 Analytical method

Total solids (TS), volatile solids (VS), total suspended solids (TSS), and volatile suspended solids (VSS) were quantified in accordance with standard methods for wastewater analysis. The pH was measured with a benchtop pH meter fitted with a combined electrode (Orion™ Versa Star Pro™, Thermo Fisher Scientific, Massachusetts, United States). Biogas composition was determined using a gas chromatograph (ACME 6100, Younglin, Anyang, Korea) coupled with a thermal conductivity detector (TCD). The concentrations of organic acids (lactic acid, formic acid, acetic acid, propionic acid, and butyric acid) and residual glucose were monitored daily. Before analysis, samples were centrifuged, and the collected supernatants were passed successively through 0.45 and 0.20 μm membrane filters. Volatile fatty acids (VFAs) were analyzed by high-performance liquid chromatography (HPLC; Waters 2487, Milford, MA, United States) equipped with a UV detector at 210 nm and an Aminex HPX-87H column for organic acid analysis (300 × 7.8 mm, Bio-Rad, United States). The mobile phase was 0.01 N H_2_SO_4_ at a flow rate of 0.6 mL/min. The column temperature was maintained at 35°C, and the injection volume was 20 μL. Residual glucose concentrations were measured using the same HPLC system equipped with a refractive index (RI) detector and an Aminex HPX-87P carbohydrate analysis column (300 × 7.8 mm, Bio-Rad, United States). Deionized water was used as the mobile phase at a flow rate of 0.6 mL/min. The column temperature was maintained at 85°C, and the injection volume was 20 μL. For all HPLC analyses, each sample was filtered through a 0.45 μm membrane filter prior to injection. All physicochemical analyses were performed in duplicate, and the results are presented as mean ± standard deviation.

### 2.5 Microbial community analysis

Granule biomass samples (1.5 mL) were collected from each reactor at the end of each HRT period and centrifuged at 13,000 × g for 1 min to obtain biomass pellets. Effluent suspended biomass was not used for DNA extraction because granular sludge represents the core biomass of UASB reactors. The pellets were stored at −75°C, and approximately 300–500 mg of wet pellet was used for genomic DNA extraction using a Soil DNA isolation kit (SG-DN-SO-L; SmartGene, Republic of Korea). The V4 region of the bacterial 16S rRNA gene was amplified using barcode-tagged primers. For bacterial amplification, the fusion primers 515F (5′-GTGCCAGCMGCC GCGGTAA-3′) and 806R (5′-GGACTACHVGGGTWT CTAAT-3′) were used, and sequencing was carried out on an Illumina iSeq 100 system (Illumina, United States). Raw sequence reads were processed using QIIME2 amplicon version 2024.10. Primer and low-quality sequences were trimmed using the cutadapt plugin. Sequence denoising was performed using the DADA2 plugin with the denoise-single command, and the resulting features were reported as amplicon sequence variants (ASVs). Taxonomic assignment was performed using the classify-consensus-blast method against the EzBioCloud 16S rRNA gene database with a minimum percent identity of 90%. Because genus-level classification grouped several key hydrogen-producing taxa within the genus Clostridium, species-level taxonomic assignment was used to track the persistence of the bioaugmented *C. pasteurianum* and to distinguish it from other dominant *Clostridium* taxa. The resulting relative abundance data were extracted for further visualization and interpretation. Associations between microbial community structure, including bacterial populations, and environmental variables in each digester were evaluated by non-metric multidimensional scaling (NMDS) based on Bray–Curtis dissimilarity using the vegan package (version 4.5.0) in R software (Jiang et al., 2021; Salmaso, 1996). Functional characteristics of the bacterial community were further inferred using PICRUSt2 (Phylogenetic Investigation of Communities by Reconstruction of Unobserved States). PICRUSt2 analysis was performed based on the representative ASV sequences and feature table exported from QIIME2. Predicted functional profiles were generated based on KEGG Orthology (KO), and the KO profiles associated with hydrogen-producing and competing metabolic pathways were used for functional interpretation (Zhang et al., 2025).

## 3 Results and discussion

### 3.1 Continuous biohydrogen production performance

**FIGURE 2:**
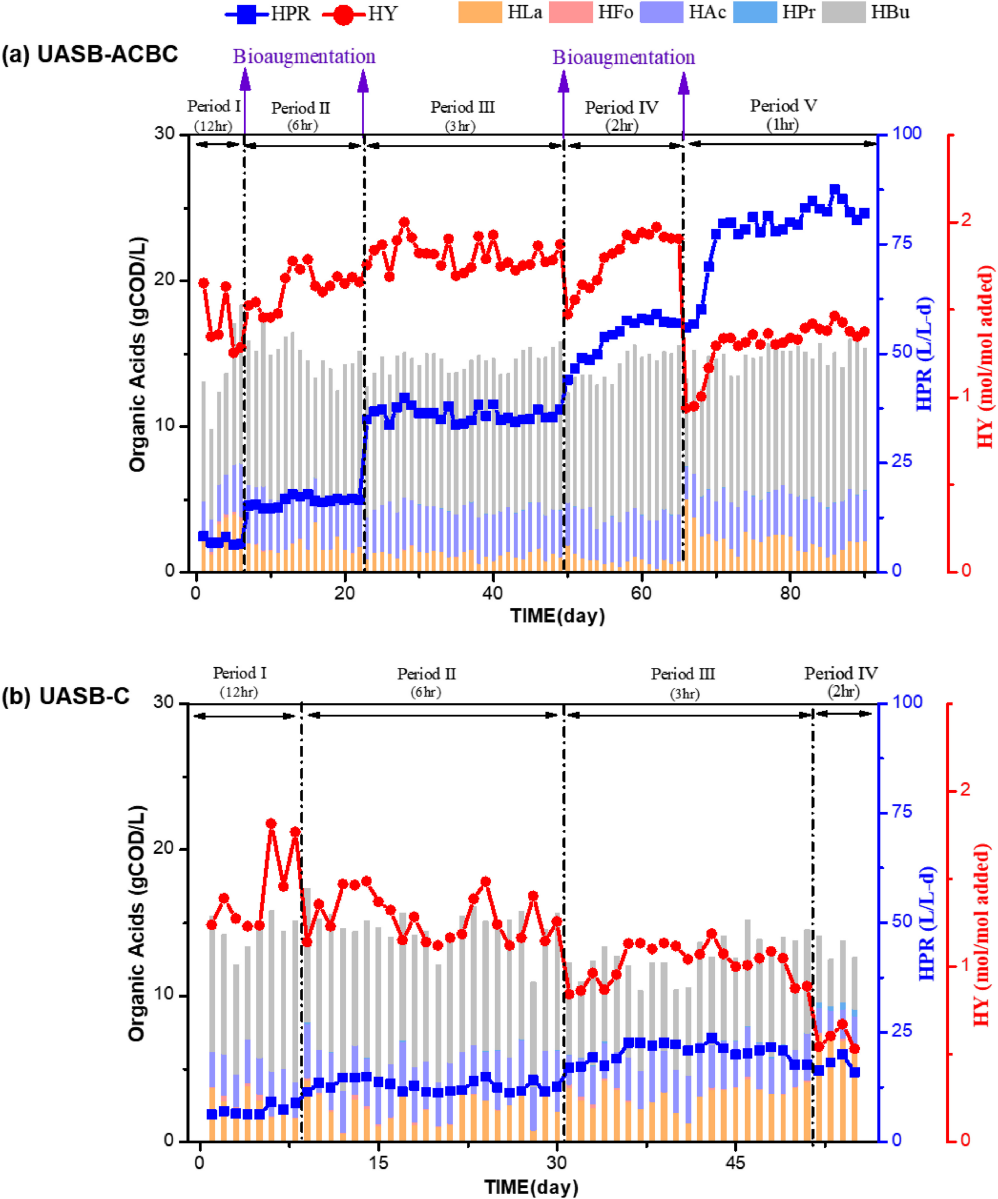
Daily continuous hydrogen yield (HY), hydrogen production rate (HPR), and organic acids (OAs) profiles in **(a)** UASB-ACBA and **(b)** UASB-C.

Continuous biohydrogen production was evaluated using UASB-C and UASB-ACBA under the operational periods described in section 2.2. Figure 2 shows the daily hydrogen yield (HY), hydrogen production rate (HPR), and organic acid concentrations. The UASB systems were initiated with a 12 h HRT. For UASB-ACBA, the average HY and HPR in Period I were 1.46 mol H_2_/mol glucose consumed and 7.08 L/L/d, respectively, which were slightly lower than those of UASB-C (Table 1). However, as the HRT decreased, both parameters increased in UASB-ACBA. The maximum HY of 1.94 mol H_2_/mol glucose consumed was observed in Period IV, where the OLR increased to 256.08 g COD/L/d while the SLR remained relatively controlled at 28.26 g COD/g VSS/d because of the increased reactor-retained VSS concentration. The further increase in HPR in Period V, despite the decrease in HY, was likely associated with the high carbon inflow rate and increased reactor-retained VSS concentration. For UASB-C, the average HY and HPR in Period I (12 h HRT) were 1.54 mol H_2_/mol glucose_consumed_ and 7.28 L/L/d, respectively. As the HRT decreased, the HY declined by 48.7%, from 1.54 (Period I) to 0.79 mol H_2_/mol glucose_consumed_ in Period IV (2 h HRT). The HPR increased until Period III (3 h HRT) and then decreased in Period IV, which corresponded well with the trend of volatile suspended solids (VSS) in the UASB-C. Because UASB-C showed a decline in both HPR and reactor-retained VSS concentration at 2 h HRT, indicating substantial biomass washout and deterioration of reactor performance, the reactor was not further operated at the 1 h HRT. Under these conditions, further shortening the HRT was considered unlikely to maintain stable hydrogen production.

The increase in volumetric OLR from 42.68 to 512.16 g COD/L/d in UASB-ACBA and from 42.68 to 256.08 g COD/L/d in UASB-C imposed progressively stronger organic loading pressure on the UASB systems (Table 1). In UASB-ACBA, although the OLR increased sharply with decreasing HRT, the SLR remained relatively controlled and even decreased from 37.85 g COD/g VSS/d in Period III to 24.19 g COD/g VSS/d in Period V because of the increased reactor-retained VSS concentration. This trend suggests that the increased reactor-retained biomass may have distributed the increased organic load across a larger retained biomass pool, which could have contributed to sustained volumetric hydrogen production under high-throughput conditions. Similar relationships between biomass retention and stable reactor performance have been reported previously (Yu and Mu, 2006; Zhang et al., 2007). In contrast, UASB-C showed a sharp increase in SLR, nearly doubling from 42.15 to 83.41 g COD/g VSS/d in Period IV because the VSS concentration decreased under the shortened HRT. The elevated SLR in UASB-C indicates that the remaining biomass was exposed to excessive substrate loading, which likely contributed to the decline in HY and HPR at 2 h HRT. Although quantitative granule size analysis was not conducted, these VSS trends suggest greater biomass accumulation in UASB-ACBA and reduced biomass retention in UASB-C under shortened HRT conditions.

A comparison between UASB-C and UASB-ACBA suggests that the combined application of GAC and bioaugmentation improved dark fermentation performance under shortened HRT conditions. Compared with the control reactor (UASB-C), UASB-ACBA achieved a 26.0% higher maximum average HY, increasing from 1.54 mol H_2_/mol glucose consumed in UASB-C to 1.94 mol H_2_/mol glucose consumed in UASB-ACBA. UASB-ACBA also showed an approximately four-fold higher maximum average HPR than UASB-C, increasing from 20.46 L/L/d in UASB-C to 83.08 L/L/d in UASB-ACBA (Table 1). In the UASB-C reactor, the HPR declined by 14.4% at 2 h HRT (from 20.46 to 17.52 L/L/d), suggesting that excessive hydraulic stress may have reduced reactor-retained biomass and hydrogen-producing activity, leading to lower hydrogen productivity. In contrast, the UASB-ACBA system maintained stable hydrogen production under similar shortened HRT conditions, indicating that the reactor was better able to withstand hydraulic and organic loading stress without a marked decline in volumetric HPR. The operation at 1 h HRT represented a highly intensified condition because the OLR increased to 512.16 g COD/L/d and the hydraulic loading was substantially increased. Under this condition, UASB-ACBA maintained high volumetric HPR, while the reactor-retained VSS concentration increased. These performance enhancements may be associated with increased reactor-retained biomass and the maintenance of hydrogen-producing populations under shortened HRT conditions. To evaluate these results in comparison with previous studies, the performance was compared with that of continuous reactors employing GAC supplementation or bioaugmentation individually (Supplementary Table 1). In a GAC-based anaerobic fluidized-bed reactor (AFBR), a maximum HPR of 56.6 L/L/d was obtained at a 1 h HRT (Zhang et al., 2007), whereas a continuous stirred-tank reactor (CSTR) bioaugmented with *C. butyricum* reached 58.57 L/L/d at a 2 h HRT (Sim et al., 2022). In the present study, the combined application of GAC and *repeated C. pasteurianum* bioaugmentation achieved a maximum HPR of 83.08 L/L/d while maintaining stable hydrogen production down to a 1 h HRT. This value was approximately 42–47% higher than those of the GAC-only and bioaugmentation-only systems Although a strict comparison is limited by differences in reactor configuration, substrate, and operating temperature, this result suggests that combining GAC with repeated *C. pasteurianum* bioaugmentation sustains higher volumetric productivity at a shorter HRT than either strategy alone, consistent with a complementary rather than redundant effect.

### 3.2 Batch hydrogen-producing activity of biomass collected during continuous operation

Table 2 and Supplementary Figure 1 summarize the hydrogen-producing activities (HPA) of UASB-C and UASB-ACBA across the operational periods. In UASB-ACBA, the lag phase did not decrease monotonically but varied across the operating periods: 39.0 h in Period II, 59.0 h in Period III, 52.1 h in Period IV, and 26.9 h in Period V. The longer lag phases in Periods III and IV may reflect temporary microbial adaptation to shortened HRT and increased organic loading, whereas the marked decrease in Period V suggests that the retained hydrogen-producing biomass became more rapidly activated under high-throughput conditions. However, the reduced HY in Period V indicates that faster activation did not necessarily correspond to higher conversion efficiency, which may indicate that part of the substrate flow was redirected toward non-hydrogenic pathways under the extremely short HRT. In UASB-C, the lag phase decreased from 93.8 h in Period II to 63.3 h in Period III but increased again to 81.5 h in Period IV, indicating delayed metabolic activation under severe hydraulic stress. The shorter lag phase observed for UASB-ACBA than for UASB-C in Periods II and IV suggests that biomass collected from UASB-ACBA had a more rapid activation potential under the batch HPA conditions, although differences in the S/I ratio among periods should also be considered when interpreting the kinetic parameters. The HY in UASB-ACBA decreased during Periods III–IV but recovered to 1.47 moL H_2_/moL glucose in Period V, suggesting that the collected biomass retained hydrogen-producing potential under the batch HPA conditions. Conversely, the HY of UASB-C gradually decreased from 1.42 to 1.16 moL H_2_/mol glucose_*added*_, suggesting that flow was partly redirected toward non-hydrogenic pathways under shortened HRT conditions. The HPR of UASB-ACBA was higher than that of UASB-C in the batch HPA tests during Periods II and III, reaching a peak of 11.85 mL H_2_/h in Period III. However, in Period IV, UASB-C showed a slightly higher batch HPR than UASB-ACBA. Therefore, the batch HPA results do not indicate consistently higher intrinsic activity of UASB-ACBA biomass across all periods.

The biomass-specific HY of UASB-ACBA decreased from 32.20 to 5.61 mL H_2_/g VSS as the VSS concentration increased, whereas UASB-C showed higher biomass-specific HY values except in Period II. This result does not indicate superior continuous reactor performance of UASB-C, because biomass-specific HY reflects hydrogen production per unit biomass under batch conditions. In UASB-ACBA, the higher retained VSS may have lowered the apparent activity per unit biomass by distributing the same substrate concentration over a larger biomass pool and by including less active biomass fractions, extracellular polymeric substances, or diffusion-limited zones. It should also be noted that the substrate-to-inoculum (S/I) ratio was not independently controlled in the HPA assays but was determined by the reactor-retained VSS concentration at the time of biomass sampling. Consequently, the substantial variation in S/I ratio among operational periods may have influenced the observed kinetic parameters, particularly the lag phase and biomass-specific hydrogen yield. Therefore, these HPA results should be interpreted together with the corresponding S/I ratios and should not be regarded solely as evidence of intrinsic changes in biomass activity.

### 3.3 Distribution of metabolic flux

Table 3 summarizes the COD balance, including fermentation by-products, residual glucose, and hydrogen produced throughout UASB operation. The COD balance ranged from 79.55 to 92.58%, suggesting that the major fermentation products were reasonably represented, although the incomplete closure indicates that some minor or unidentified products may not have been fully included in the balance. The lower COD balance closure in UASB-ACBA does not necessarily indicate poorer reactor performance. In this study, COD balance was calculated based on measured soluble metabolites, residual glucose, and produced hydrogen, whereas biomass-associated COD, extracellular polymeric substances, GAC-associated organic matter, and unidentified minor metabolites were not fully included. Because UASB-ACBA showed substantial reactor-retained VSS accumulation, part of the influent COD may have been incorporated into retained biomass or EPS, resulting in lower apparent COD recovery. In contrast, the higher COD balance in UASB-C may reflect the greater accumulation of measured soluble products, such as lactate and residual glucose, rather than better hydrogen-producing performance. In the UASB-ACBA system, butyric acid (HBu) was the dominant byproduct throughout all operating periods, followed by acetic acid (HAc). The residual glucose concentration remained within 0.38–1.38 g COD/L, suggesting that the system generally maintained hydrogen fermentation with efficient substrate utilization even under shortened HRT conditions. UASB-C showed a marked shift in product distribution at Period IV, where lactic acid increased to 7.08 g COD/L and 5.53 g COD/L of glucose remained in the effluent. Rather than indicating simple process instability, this pattern suggests the establishment of a lactate-prevalent metabolic state with incomplete substrate conversion under the intensified loading condition. The shortened HRT and increased organic loading may have promoted lactate-associated fermentation. However, lactate production itself should not be interpreted as inherently unfavorable for hydrogen production, because lactate can act as an intermediate substrate in lactate-driven hydrogen production. Under balanced conditions, lactate may be consumed together with acetate by lactate-fermenting hydrogen-producing bacteria to produce butyrate and hydrogen. Therefore, the lactate accumulation observed in UASB-C is better interpreted as a possible imbalance between lactate formation and lactate reutilization, rather than as direct evidence that lactate production inhibited hydrogen generation (Montoya-Rosales et al., 2023).

**TABLE 3:** Chemical oxygen demand (COD) balance of metabolites, residual glucose, and hydrogen produced within the systems.

Reactor	Period	HRT (h)	By-product in effluent (g COD/L)					Remained glucose (g COD/L)	Produced hydrogen (g COD/L)	COD balance (g COD/L)
			HLa	HFo	HAc	HPr	HBu			
UASB-ACBA	I	12	3.02 ± 1.08	0.20 ± 0.02	2.82 ± 0.52	0.00 ± 0.00	8.01 ± 1.94	0.45 ± 0.39	2.53 ± 0.31	17.02 (79.55%)
	II	6	1.94 ± 0.64	0.04 ± 0.01	3.24 ± 0.33	0.01 ± 0.01	9.20 ± 1.21	1.20 ± 0.74	3.00 ± 0.11	18.63 (87.04%)
	III	3	1.16 ± 0.29	0.01 ± 0.01	3.14 ± 0.13	0.03 ± 0.01	10.46 ± 0.76	0.38 ± 0.29	3.20 ± 0.12	18.37 (85.85%)
	IV	2	0.69 ± 0.25	0.00 ± 0.00	3.25 ± 0.15	0.02 ± 0.02	10.83 ± 0.70	0.38 ± 0.40	3.39 ± 0.08	18.56 (86.72%)
	V	1	1.69 ± 0.40	0.00 ± 0.00	3.40 ± 0.19	0.03 ± 0.03	10.06 ± 0.55	1.38 ± 0.24	2.47 ± 0.07	19.08 (89.15%)
UASB-C	I	12	2.39 ± 0.85	0.15 ± 0.12	2.69 ± 0.36	0.00 ± 0.00	9.10 ± 1.86	0.98 ± 0.54	2.60 ± 0.48	17.90 (83.66%)
	II	6	2.45 ± 0.89	0.03 ± 0.04	3.48 ± 0.51	0.03 ± 0.03	8.81 ± 0.69	2.05 ± 0.36	2.23 ± 0.22	19.09 (89.23%)
	III	3	3.62 ± 0.37	0.09 ± 0.05	3.10 ± 0.40	0.05 ± 0.03	6.85 ± 0.58	4.23 ± 0.83	1.83 ± 0.16	19.77 (92.40%)
	IV	2	7.08 ± 0.43	0.13 ± 0.06	1.78 ± 0.25	0.41 ± 0.07	3.84 ± 0.61	5.53 ± 0.90	1.04 ± 0.12	19.81 (92.58%)

Metabolite, residual glucose, and hydrogen values are presented as mean ± standard deviation of triplicate measurements for each operating condition. COD balance values were calculated from the corresponding mean values.

Based on the stoichiometric balance, acetate-type fermentation yields a maximum of 4 mol of hydrogen per mol of glucose, whereas the butyrate-type pathway produces only 2 mol [Equations 2,3] (Logan, 2004),


$$C_{6}\,H_{12}\,O_{6}+2\,H_{2}\,O\to 2\,C_{2}\,H_{4}\,O_{2}+2\,C\,O_{2}+4\,H_{2}$$ (2)

$$C_{6}\,H_{12}\,O_{6}\to C_{4}\,H_{8}\,O_{2}+2\,C\,O_{2}+2\,H_{2}$$ (3)

However, despite its higher theoretical potential, the acetate pathway does not always result in greater hydrogen production because a portion of the hydrogen can be reutilized through the homoacetogenic reaction, which converts hydrogen and carbon dioxide into acetate [Equation 4] (Saady, 2013),


$$2\,C\,O_{2}+4\,H_{2}\to C_{2}\,H_{4}\,O_{2}+2\,H_{2}\,O$$ (4)

Furthermore, the formation of propionate (HPr) and formate (HFo) represent additional hydrogen-consuming routes that compete with hydrogen generation [Equations 5 and 6] (Angenent et al., 2004),


$$C_{6}\,H_{12}\,O_{6}+2\,H_{2}\to 2\,C_{3}\,H_{6}\,O_{2}+2\,H_{2}\,O$$ (5)

$$C\,O_{2}+H_{2}\to C\,H_{2}\,O_{2}$$ (6)

The apparent contribution of hydrogen-consuming routes can be estimated using a stoichiometric flux balance based on the difference between theoretical and measured hydrogen yields (Kim et al., 2006; Lalman et al., 2013). Accordingly, the theoretical hydrogen yield was calculated from the substrate consumption determined through metabolite analysis [Equation 7] (Kim et al., 2006), respectively,


$$T\,h\,e\,o\,r\,e\,t\,i\,c\,a\,l\,h\,y\,d\,r\,o\,g\,e\,n\,y\,i\,e\,l\,d\,\left(\frac{m\,o\,l\,H_{2}}{m\,o\,l\,g\,l\,u\,c\,o\,s\,e_{\text{consumed}}}\right)$$ (7)

$$\;=\left(\frac{2\,m\,o\,l\,H_{2}}{m\,o\,l\,b\,u\,t\,y\,r\,a\,t\,e}\times \frac{m\,o\,l\,b\,u\,t\,y\,r\,a\,t\,e}{m\,o\,l\,g\,l\,u\,c\,o\,s\,e_{\text{consumed}}}\right)$$

$$\;+\left(\frac{2\,m\,o\,l\,H_{2}}{m\,o\,l\,a\,c\,e\,t\,a\,t\,e}\times \frac{m\,o\,l\,a\,c\,e\,t\,a\,t\,e}{m\,o\,l\,g\,l\,u\,c\,o\,s\,e_{\text{consumed}}}\right)$$

$$\;\;-[\left(\frac{m\,o\,l\,H_{2}}{m\,o\,l\,p\,r\,o\,p\,i\,o\,n\,a\,t\,e}\times \frac{m\,o\,l\,p\,r\,o\,p\,i\,o\,n\,a\,t\,e}{m\,o\,l\,g\,l\,u\,c\,o\,s\,e_{\text{consumed}}}\right)$$

$$\;+\left(\frac{m\,o\,l\,H_{2}}{m\,o\,l\,f\,o\,r\,m\,a\,t\,e}\times \frac{m\,o\,l\,f\,o\,r\,m\,a\,t\,e}{m\,o\,l\,g\,l\,u\,c\,o\,s\,e_{\text{consumed}}}\right)]$$

**FIGURE 3:**
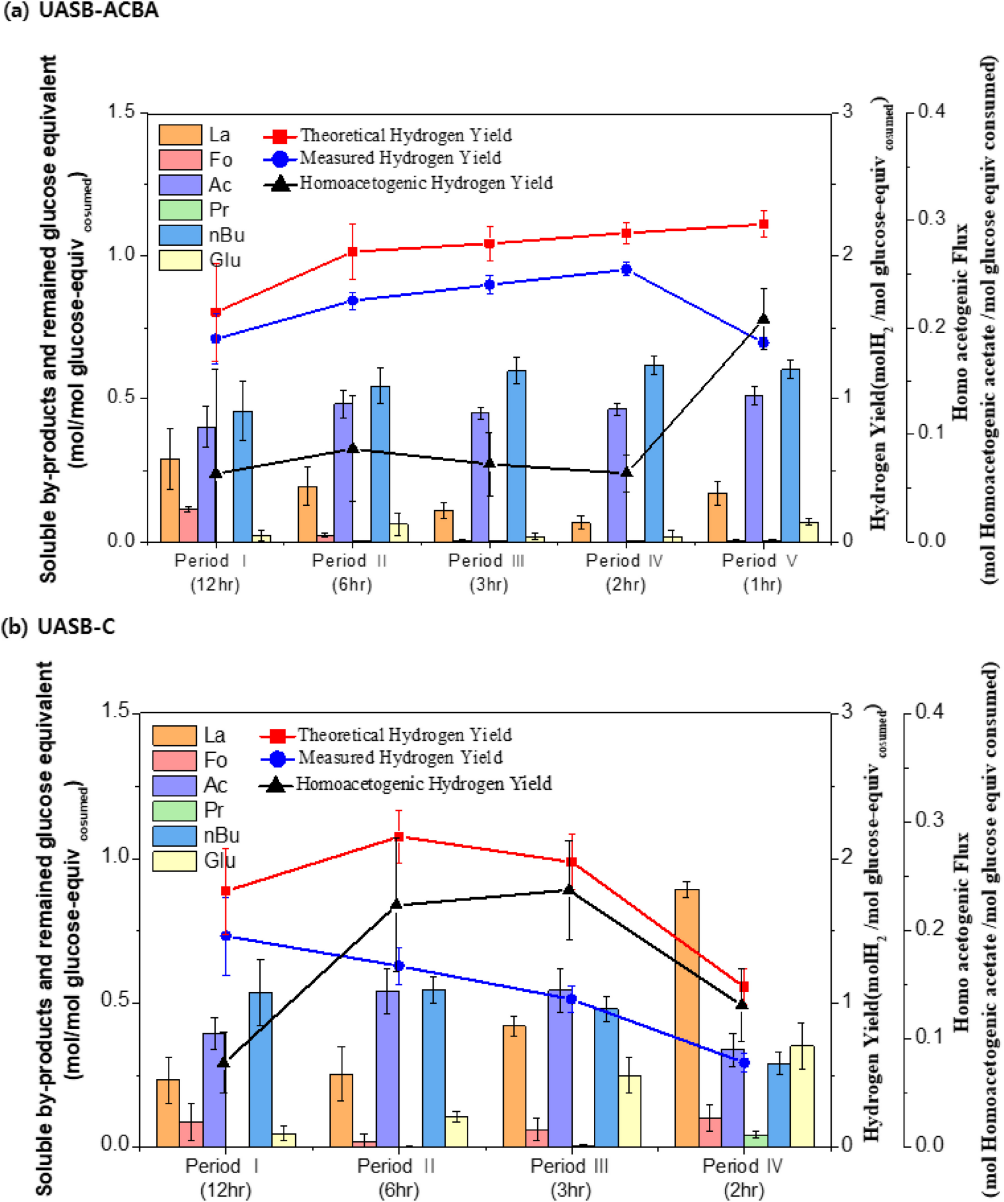
Comparison of experimental and theoretical hydrogen yields, homoacetogenic pathway fluxes, and metabolic by-products across operational periods in **(a)** UASB-ACBA and **(b)** UASB-C. Values were calculated from the mean values of triplicate measurements for each operating condition.

Figure 3 presents a comparison between the measured and theoretical hydrogen yields, together with the apparent homoacetogenic contribution and corresponding by-product distribution in each period. In UASB-ACBA, the gap between the theoretical and measured HY remained relatively small from Periods I to IV, where the measured HY was 11.5–17.0% lower than the theoretical value. During the same periods, butyrate remained the dominant soluble product, and effluent lactate decreased from 3.02 to 0.69 g COD/L (Table 3). These results suggest that UASB-ACBA suggests a relatively balanced distribution of reducing equivalents toward hydrogen- and butyrate-associated pathways under shortened HRT conditions. However, this pattern should not be interpreted as direct suppression of lactate-associated fermentation or homoacetogenesis, because recent metatranscriptomic evidence indicates that lactate-type fermentation, homoacetogenesis, and hydrogen-producing pathways can coexist in high-rate dark fermentation systems without necessarily compromising H_2_ production (Montoya-Rosales et al., 2023).

In Period V, the measured HY of UASB-ACBA decreased and became 37.4% lower than the theoretical HY, accompanied by an increase in the estimated homoacetogenic contribution and effluent lactate concentration. This suggests that the extremely shortened HRT of 1 h may have partially disturbed the balance between lactate formation and lactate reutilization, although high volumetric HPR was still maintained. In UASB-C, the measured HY remained 41.6–58.0% lower than the theoretical value from Periods II to IV, while effluent lactate increased from 2.45 to 7.08 g COD/L and residual glucose reached 5.53 g COD/L at Period IV (Table 3). This pattern suggests a shift toward a lactate-prevalent metabolic state with incomplete substrate conversion under intensified loading conditions. Therefore, the lower hydrogen production performance of UASB-C is better interpreted as a possible imbalance in lactate-associated fermentation, rather than as evidence that lactate production itself inhibited hydrogen generation.

Overall, the metabolic flux analysis suggests that UASB-ACBA maintained a more balanced lactate-associated fermentation pattern under progressively shortened HRT conditions. This interpretation is consistent with previous reports but should be considered hypothetical because metabolic flux was inferred from stoichiometric analysis rather than directly measured (García-Depraect et al., 2021; Regueira-Marcos et al., 2023). The difference between theoretical and measured HY should be interpreted cautiously as an indication that part of the reducing equivalents was not recovered as hydrogen, potentially due to coexisting reductive routes, biomass-associated COD, extracellular polymeric substances, unidentified minor products, and lactate-associated intermediates. Thus, lactate production and homoacetogenesis should not be described only as unfavorable or competing pathways, but as coexisting metabolic routes whose effects on hydrogen production depend on the balance between substrate conversion, lactate formation, lactate reutilization, and hydrogen recovery.

### 3.4 Microbial community analysis

#### 3.4.1 Impact of bioaugmentation on microbial community

**FIGURE 4:**
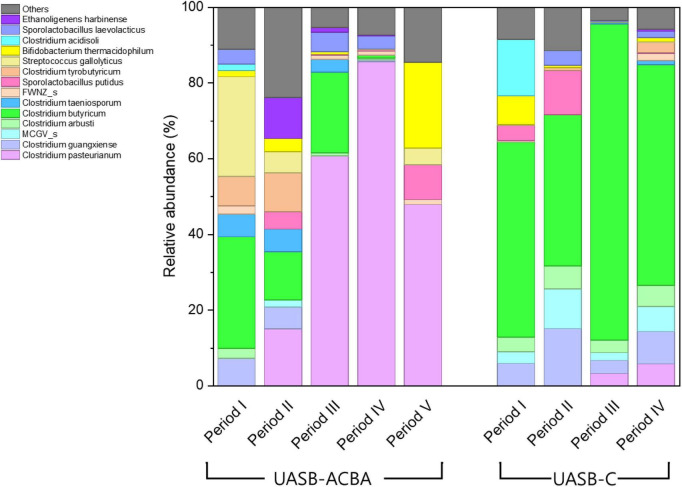
Taxonomic composition of microbial communities at the lowest assignable taxonomic level during each operational period. Species-level names, where shown, represent putative assignments based on 16S rRNA gene sequence matching and were used to distinguish the bioaugmented *C. pasteurianum* from other dominant *Clostridium* taxa.

Figure 4 shows the taxonomic composition of microbial communities at the lowest assignable taxonomic level during each operational period, where “Others” represents the sum of microbial species whose total relative abundance across all samples was < 5% of the total microbiome. The microbiome was analyzed using granule biomass samples collected from each reactor at the end of each HRT period, rather than suspended biomass. Therefore, the microbial community results represent the granule-associated reactor biomass, which is the core biomass of the UASB system. In the UASB-ACBA system, *Clostridium butyricum* was dominant in Period I, followed by *Streptococcus gallolyticus*, a carbohydrate-fermenting bacterium capable of utilizing a wide range of carbohydrates (Chamkha et al., 2002). The presence of carbohydrate-fermenting bacteria may facilitate the conversion of carbohydrates into central metabolic intermediates, which are subsequently utilized for the production of various fermentation products, including lactate, acetate, and butyrate (Show et al., 2012) Therefore, the coexistence of *S. gallolyticus* with lactate accumulation suggests its possible involvement in carbohydrate fermentation associated with lactate production in the present system. However, the presence of lactic acid-producing bacteria should not be interpreted only as unfavorable competition, because lactate can serve as an intermediate substrate for lactate-fermenting hydrogen-producing Clostridium species (Fujita et al., 2010). In Period II, bioaugmentation led to the coexistence of various hydrogen-producing species, whereas in Period III, The ASV assigned to *Clostridium pasteurianum* became the dominant hydrogen-producing taxon, accounting for 60.7% of total abundance. Following repeated bioaugmentation at each HRT transition, the ASV assigned to *Clostridium pasteurianum* increased in relative abundance compared with other dominant *Clostridium* taxa during Periods III and IV. Because *C. pasteurianum* was repeatedly introduced, this increase should be interpreted as the persistence and relative dominance of the introduced population under the tested reactor conditions, rather than as evidence that continuous bioaugmentation created favorable reactor conditions. The relative abundance of *Clostridium pasteurianum* further increased to 85.6% in Period IV but decreased to 47.9% in Period V, likely due to it being washed out in relation to the excessively short HRT. This condition also appeared to promote the relative enrichment of lactic acid–producing bacteria such as *Bifidobacterium thermacidophilum* (22.7%) (Seon et al., 2014; Zhu et al., 2003). In contrast, in the UASB-C reactor, *Clostridium acidisoli* accounted for 15.0% of the relative abundance in Period I. *C. acidisoli* has been reported as an acid-tolerant anaerobic bacterium isolated from acidic environments (Kuhner et al., 2000). In addition, *Sporolactobacillus*, a lactic acid producer, was detected in all periods except Period III (Zhao et al., 2010), suggesting that lactate-associated fermentation was also present in the control reactor. *Clostridium butyricum* was dominant throughout all periods, ranging from 40.0 to 83.5%, with the highest relative abundance (83.5%) observed in Period III, which corresponded to the highest HPR. These results indicate that *Clostridium butyricum* made a substantial contribution to the hydrogen production performance of UASB-C. The detection and dominance of *C. pasteurianum* in granule biomass after repeated bioaugmentation suggest that the introduced strain was retained within the UASB biomass, supporting the relevance of the UASB configuration for maintaining hydrogen-producing populations under shortened HRT conditions. However, because GAC-attached biomass was not separately analyzed, the specific distribution of *C. pasteurianum* between granules and GAC-associated biofilms could not be determined. The coexistence of ASVs assigned to *C. butyricum, C. pasteurianum*, and lactic acid-producing bacteria suggests a possible lactate-associated microbial network in the UASB systems. In such a network, lactic acid-producing bacteria may produce lactate from glucose-derived intermediates, whereas lactate-fermenting hydrogen-producing Clostridium species may reutilize lactate together with acetate to produce butyrate and hydrogen. This proposed ecological interaction is inferred from taxonomic composition and should be validated using functional analyses such as meta transcriptomics. Therefore, the detection of lactic acid-producing bacteria in this study should be interpreted as part of a potential lactate-driven hydrogen-producing community rather than as direct evidence of inhibition of hydrogen production. Because this interpretation is based on 16S rRNA gene sequencing, it should be considered an ecological explanation rather than direct evidence of active lactate conversion.

#### 3.4.2 Predicted functional potential analysis

**FIGURE 5:**
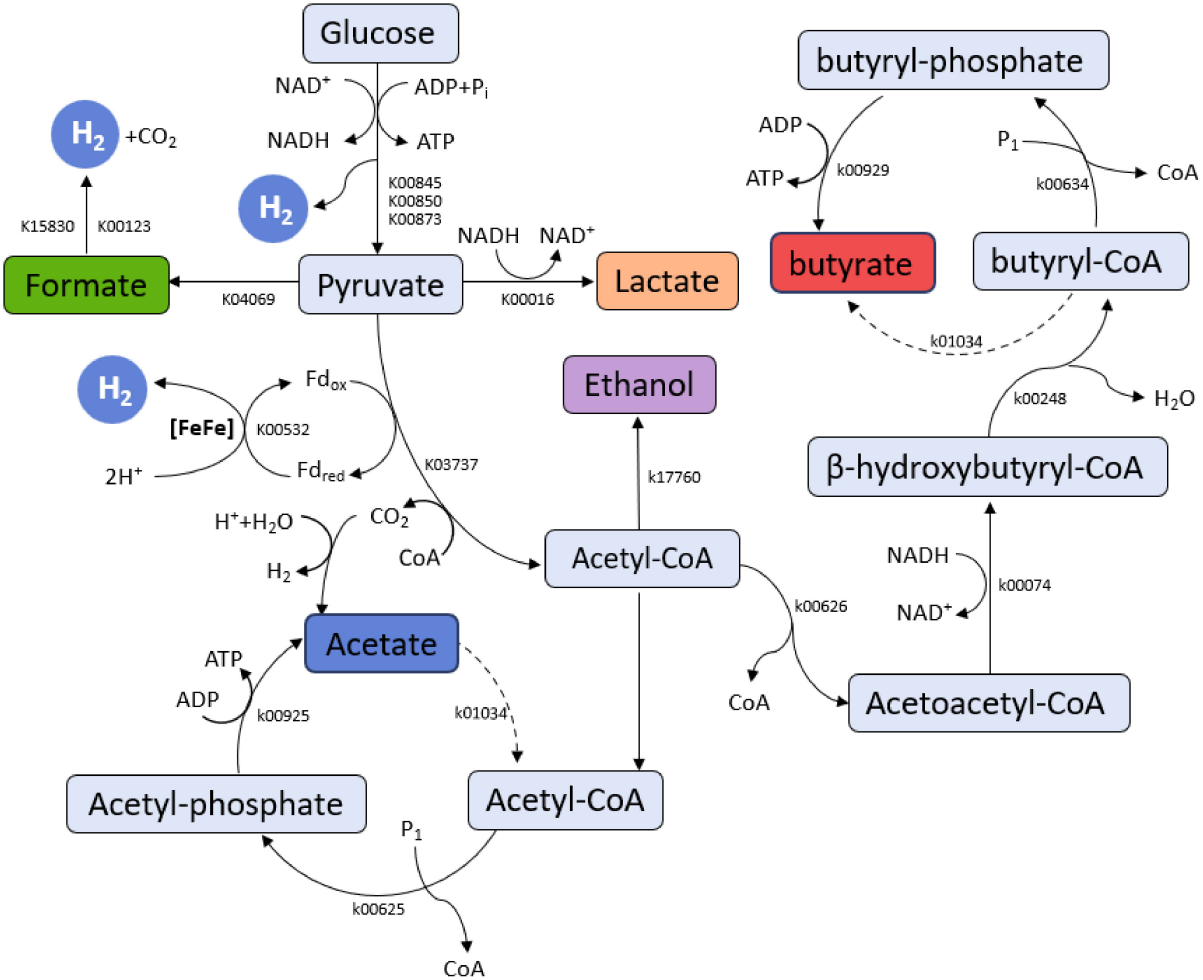
Diagram of glucose metabolic pathway associated with hydrogen production.

**FIGURE 6:**
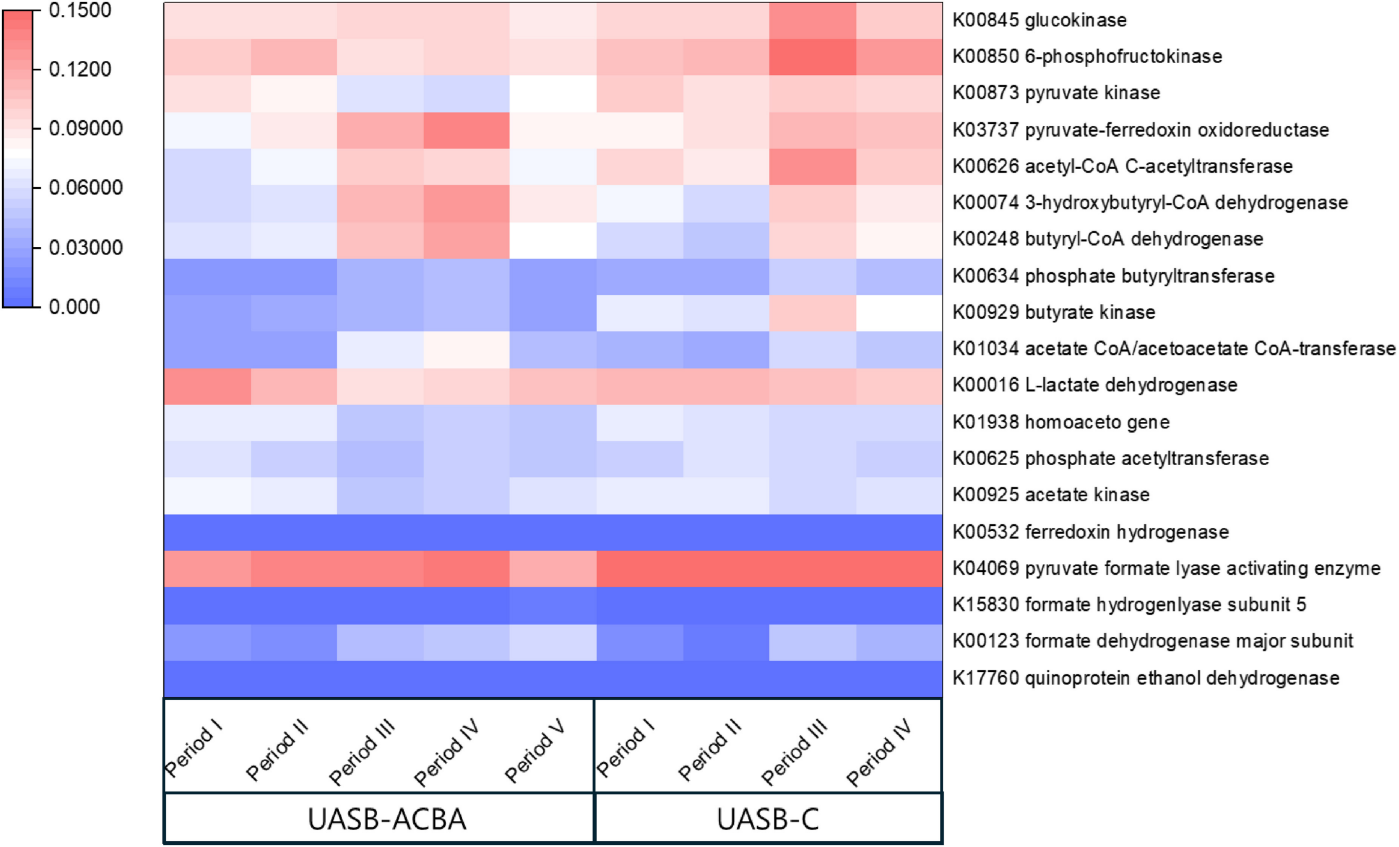
Predicted functional gene profiles associated with fermentative hydrogen production.

This study employed the KEGG database, which reportedly covers approximately 85–90% of microbial functional annotations (Park et al., 2020). Accordingly, PICRUSt2 analysis using KEGG references was used to infer the predicted functional potential of the microbial community. Because PICRUSt2 predicts functional profiles based on 16S rRNA gene-derived taxonomic information and reference genomes, the results indicate predicted genomic potential, such as the potential presence or relative abundance of metabolic pathways, rather than actual gene expression, enzyme activity, or metabolic flux (Yin and Wang, 2021). This study focuses on the glucose metabolic pathway associated with hydrogen production (Figure 5). According to the predicted functional profile shown in Figure 6, the genes involved in glycolysis and pyruvate oxidation, including K00845 (glucokinase), K00850 (6-phosphofructokinase), K00873 (pyruvate kinase), and K03737 (pyruvate-ferredoxin oxidoreductase), were highly abundant in most samples (VanFossen et al., 2009). This suggests that the microbial community had a high predicted potential for substrate oxidation through glycolysis and conversion of pyruvate to acetyl-CoA. Genes associated with the conversion of acetyl-CoA to butyryl-CoA, such as K00626 (acetyl-CoA C-acetyltransferase), K00074 (3-hydroxybutyryl-CoA dehydrogenase), and K00248 (butyryl-CoA dehydrogenase) were also maintained at relatively high levels (Sarma et al., 2017). This suggests a high predicted potential for the conversion of acetyl-CoA to butyryl-CoA, forming a sufficient pool of butyryl-CoA as a precursor for butyrate synthesis (Bennett and Rudolph, 1995). The genes involved in glycolysis and pyruvate oxidation, as well as those involved in the conversion of acetyl-CoA to butyryl-CoA, were present in relatively high abundances in Periods III, IV, and V, which is consistent with the observed enhancement of hydrogen production performance under shortened HRT conditions. In contrast, K00634 (phosphate butyryltransferase) and K00929 (butyrate kinase), which function as terminal enzymes in the classical butyrate formation pathway (phosphate butyryltransferase–butyrate kinase, ptb/buk pathway), were present in low abundances in most samples (Bao et al., 2020; Liu et al., 2020). These enzymes catalyze the conversion of butyryl-CoA to butyrate, generating intracellular ATP through substrate-level phosphorylation (Hackmann and Firkins, 2015). However, despite the low abundances of these two enzymes, the butyrate concentrations remained high. Butyrate may have been produced not only via the traditional ptb/buk pathway but also through an alternative route involving K01034 (acetate CoA/acetoacetate CoA-transferase). This interpretation is also compatible with lactate-associated butyrate formation. In lactate-driven hydrogen production, lactate-derived intermediates can be converted together with acetate toward butyrate and hydrogen formation by lactate-fermenting hydrogen-producing bacteria. Therefore, the predicted maintenance of acetyl- CoA-, butyryl- CoA-, and butyrate-related functions is consistent with the hypothesis for lactate-associated hydrogen and butyrate production in the UASB systems.

#### 3.4.3 Process parameters and microbial community correlations

**FIGURE 7:**
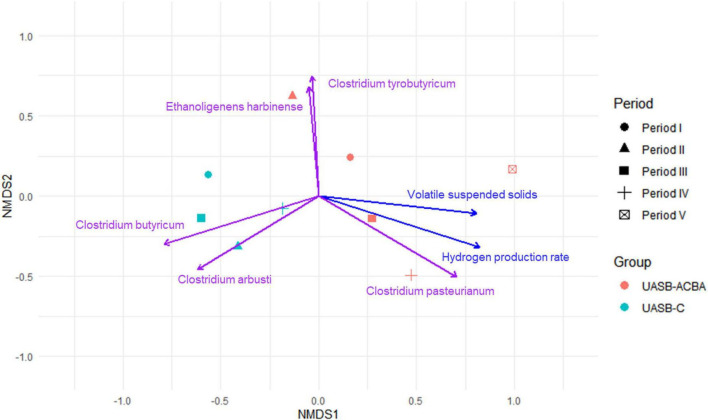
Non-metric multidimensional scaling (NMDS) plots showing the relationships between microbial community structures and environmental variables for each operational condition. Environmental variables (blue arrows) and significant microbial taxa (purple arrows) were fitted based on Bray–Curtis dissimilarity. The shape of the points represents operational period (I–V), and the color indicates reactor group.

The NMDS ordinations, based on 16S rRNA gene sequencing data, were used to visually represent the bacterial community structures under UASB-C and UASB-ACBA (Figure 7). The final NMDS stress value of 0.094 confirmed the high ordination quality, and the non-metric fit (*R*^2^ = 0.991) and linear fit (*R*^2^ = 0.945) (Supplementary Figure 2) further supported the strong goodness of fit of the NMDS results (Oksanen et al., 2026). Environmental vectors, such as hydrogen production rate and volatile suspended solids, were strongly aligned along the NMDS1 axis, indicating an association between bacterial community composition and biomass activity-related process variables. Among the bacterial taxa, *Clostridium pasteurianum* (*r*^2^ = 0.57, *p* = 0.015) and *Clostridium butyricum* (*r*^2^ = 0.51, *p* = 0.032) were moderately but significantly correlated with the ordination axes, suggesting that these hydrogen-producing Clostridium species were positively associated with hydrogen generation under the tested conditions. In contrast, *Clostridium arbusti* and *Clostridium tyrobutyricum* showed weaker associations (*r*^2^ ≈ 0.3, *p* > 0.05), suggesting that their relative abundance was less strongly associated with the observed community-level variation under the tested conditions. Collectively, these patterns suggest that the combined application of GAC and bioaugmentation was associated with the persistence of the ASV assigned to *C. pasteurianum* and with microbial community shifts related to hydrogen production under shortened HRT conditions.

Compared with previous studies that applied additive supplementation or bioaugmentation separately, the present results suggest that the combined application of GAC and *C. pasteurianum* bioaugmentation may provide complementary benefits under shortened HRT conditions. Additive-based strategies using GAC, biochar, activated carbon, or other carbonaceous supports have been reported to improve hydrogen production by enhancing microbial attachment and biomass retention (Jamali et al., 2016; Lutpi et al., 2015). In contrast, bioaugmentation-based strategies aim to reinforce specific hydrogen-producing populations, such as Clostridium species, and support the establishment of hydrogen-producing microbial communities (Mohan et al., 2005). In the present study, GAC may have provided attachment sites for biomass retention, whereas repeated *C. pasteurianum* bioaugmentation introduced and helped maintain hydrogen-producing populations under shortened HRT conditions.

The NMDS results should also be interpreted in the context of lactate-associated fermentation. The association of *C. pasteurianum* and *C. butyricum* with hydrogen production-related variables, together with the detection of lactic acid-producing bacteria and the observed lactate/butyrate patterns, is consistent with the coexistence of hydrogen-producing Clostridium species and lactate-producing bacteria. This community structure may support lactate-associated hydrogen production when lactate formation and lactate reutilization are balanced.

## 4 Conclusion

This study evaluated the combined application of granular activated carbon and *Clostridium pasteurianum* bioaugmentation in a UASB dark fermentation system operated under progressively shortened hydraulic retention times. Compared with the control reactor, the modified reactor achieved a 26% higher maximum hydrogen yield and an approximately four- fold higher hydrogen production rate, which was associated with increased reactor-retained biomass and the persistence of hydrogen-producing Clostridium populations. The metabolic results should be interpreted in the context of lactate-associated dark fermentation. Lactate production itself does not necessarily indicate reduced hydrogen production. In the control reactor, lactate, acetate, and residual glucose accumulation under shortened hydraulic retention times suggested a possible imbalance between lactate formation and lactate reutilization, whereas the modified reactor appeared to maintain a more balanced distribution of intermediates toward hydrogen- and butyrate-associated pathways. Although these interpretations are supported by previous studies and are consistent with the present observations, the proposed mechanisms should be regarded as hypotheses requiring further validation through targeted functional analyses and reactor configurations capable of distinguishing the individual effects of GAC and bioaugmentation.

## Data Availability

The original contributions presented in this study are included in the article/Supplementary material, further inquiries can be directed to the corresponding author.

## References

[B1] Ács N. Bagi Z. Rákhely G. Minárovics J. Nagy K. Kovács K. (2015). Bioaugmentation of biogas production by a hydrogen-producing bacterium. *Bioresour. Technol.* 186 286–293. 10.1016/J.BIORTECH.2015.02.098 25836037

[B2] Alexandropoulou M. Antonopoulou G. Lyberatos G. (2018). A novel approach of modeling continuous dark hydrogen fermentation. *Bioresour. Technol.* 250 784–792. 10.1016/J.BIORTECH.2017.12.005 29245129

[B3] Angenent L. Karim K. Al-Dahhan M. Wrenn B. Domíguez-Espinosa R. (2004). Production of bioenergy and biochemicals from industrial and agricultural wastewater. *Trends Biotechnol.* 22 477–485. 10.1016/j.tibtech.2004.07.001 15331229

[B4] Baeyens J. Zhang H. Nie J. Appels L. Dewil R. Ansart R. et al. (2020). Reviewing the potential of bio-hydrogen production by fermentation. *Renewable Sustainable Energy Rev.* 131:110023. 10.1016/J.RSER.2020.110023

[B5] Bao T. Feng J. Jiang W. Fu H. Wang J. Yang S. (2020). Recent advances in n-butanol and butyrate production using engineered Clostridium tyrobutyricum. *World J. Microbiol. Biotechnol.* 36 1–14. 10.1007/s11274-020-02914-2 32794091

[B6] Bennett G. Rudolph F. (1995). The central metabolic pathway from acetyl-CoA to butyryl-CoA in Clostridium acetobutylicum. *FEMS Microbiol. Rev.* 17 241–249. 10.1111/J.1574-6976.1995.TB00208.X

[B7] Carrillo-Reyes J. Celis L. Alatriste-Mondragón F. Razo-Flores E. (2012). Different start-up strategies to enhance biohydrogen production from cheese whey in UASB reactors. *Int. J. Hydrogen Energy* 37 5591–5601. 10.1016/J.IJHYDENE.2012.01.004

[B8] Carrillo-Reyes J. Celis L. Alatriste-Mondragón F. Montoya L. Razo-Flores E. (2014). Strategies to cope with methanogens in hydrogen producing UASB reactors: Community dynamics. *Int. J. Hydrogen Energy* 39 11423–11432. 10.1016/J.IJHYDENE.2014.05.099

[B9] Chamkha M. Patel B. Traore A. Garcia J. Labat M. (2002). Isolation from a shea cake digester of a tannin-degrading Streptococcus gallolyticus strain that decarboxylates protocatechuic and hydroxycinnamic acids, and emendation of the species. *Int. J. Syst. Evol. Microbiol.* 52 939–944. 10.1099/00207713-52-3-939 12054261

[B10] Chang F. Lin C. (2004). Biohydrogen production using an up-flow anaerobic sludge blanket reactor. *Int. J. Hydrogen Energy* 29 33–39. 10.1016/S0360-3199(03)00082-X

[B11] Cheng J. Li H. Ding L. Zhou J. Song W. Li Y. et al. (2020). Improving hydrogen and methane co-generation in cascading dark fermentation and anaerobic digestion: The effect of magnetite nanoparticles on microbial electron transfer and syntrophism. *Chem. Eng. J.* 397:125394. 10.1016/J.CEJ.2020.125394

[B12] Eroglu E. Melis A. (2011). Photobiological hydrogen production: Recent advances and state of the art. *Bioresour. Technol.* 102 8403–8413. 10.1016/J.BIORTECH.2011.03.026 21463932

[B13] Feng D. Xia A. Wang X. Huang Y. Zhu X. Zhu X. et al. (2024). Numerical modelling of interspecies electron transfer in anaerobic digestion using multiple electron donors for methane production. *Chem. Eng. J.* 497:154876. 10.1016/J.CEJ.2024.154876

[B14] Fujita R. Mochida K. Kato Y. Goto K. (2010). Sporolactobacillus putidus sp. nov., an endospore-forming lactic acid bacterium isolated from spoiled orange juice. *Int J. Syst. Evol. Microbiol.* 60 1499–1503. 10.1099/ijs.0.002048-0 19684317

[B15] García-Depraect O. Muñoz R. Rodríguez E. Rene E. León-Becerril E. (2021). Microbial ecology of a lactate-driven dark fermentation process producing hydrogen under carbohydrate-limiting conditions. *Int. J. Hydrogen Energy* 46 11284–11296. 10.1016/j.ijhydene.2020.08.209

[B16] Hackmann T. Firkins J. (2015). Electron transport phosphorylation in rumen butyrivibrios: Unprecedented ATP yield for glucose fermentation to butyrate. *Front. Microbiol.* 6:147018. 10.3389/fmicb.2015.00622 26157432 PMC4478896

[B17] Han W. Liu Z. Fang J. Huang J. Zhao H. Li Y. (2016). Techno-economic analysis of dark fermentative hydrogen production from molasses in a continuous mixed immobilized sludge reactor. *J. Clean. Prod.* 127 567–572. 10.1016/J.JCLEPRO.2016.04.055

[B18] Hung C. Lee K. Cheng L. Huang Y. Lin P. Chang J. (2007). Quantitative analysis of a high-rate hydrogen-producing microbial community in anaerobic agitated granular sludge bed bioreactors using glucose as substrate. *Appl. Microbiol. Biotechnol. 2007 75:3* 75 693–701. 10.1007/S00253-007-0854-7 17440720

[B19] Jamali N. Md Jahim J. Wan Isahak W. (2016). Biofilm formation on granular activated carbon in xylose and glucose mixture for thermophilic biohydrogen production. *Int. J. Hydrogen Energy* 41 21617–21627. 10.1016/J.IJHYDENE.2016.05.092

[B20] Jiang H. Chen Y. Hu Y. Wang Z. Lu X. (2021). Soil bacterial communities and diversity in alpine grasslands on the tibetan plateau based on 16S rRNA gene sequencing. *Front. Ecol. Evol.* 9:630722. 10.3389/fevo.2021.630722

[B21] Jung K. Kim D. Kim S. Shin H. (2011). Bioreactor design for continuous dark fermentative hydrogen production. *Bioresour. Technol.* 102 8612–8620. 10.1016/J.BIORTECH.2011.03.056 21489782

[B22] Kim D. Han S. Kim S. Shin H. (2006). Effect of gas sparging on continuous fermentative hydrogen production. *Int. J. Hydrogen Energy* 31 2158–2169. 10.1016/J.IJHYDENE.2006.02.012

[B23] Kuhner C. Matthies C. Acker G. Schmittroth M. Gößner A. Drake H. (2000). Clostridium akagii sp. nov. and Clostridium acidisoli sp. nov.: Acid- tolerant, N2-fixing clostridia isolated from acidic forest soil and litter. *Int. J. Syst. Evol. Microbiol.* 50 873–881. 10.1099/00207713-50-2-873 10758899

[B24] Kumar G. Bakonyi P. Kobayashi T. Xu K. Sivagurunathan P. Kim S. et al. (2016). Enhancement of biofuel production via microbial augmentation: The case of dark fermentative hydrogen. *Renewable Sustainable Energy Rev.* 57 879–891. 10.1016/J.RSER.2015.12.107

[B25] Lalman J. Chaganti S. Moon C. Kim D. (2013). Elucidating acetogenic H2 consumption in dark fermentation using flux balance analysis. *Bioresour. Technol.* 146 775–778. 10.1016/J.BIORTECH.2013.07.125 23958339

[B26] Lin C. Chiang C. Thi Nguyen M. Lay C. (2017). Enhancement of fermentative biohydrogen production from textile desizing wastewater via coagulation-pretreatment. *Int. J. Hydrogen Energy* 42 12153–12158. 10.1016/J.IJHYDENE.2017.03.184

[B27] Liu B. Popp D. Müller N. Sträuber H. Harms H. Kleinsteuber S. (2020). Three novel clostridia isolates produce n-Caproate and iso-butyrate from lactate: Comparative genomics of chain-elongating bacteria. *Microorganisms* 8:1970. 10.3390/MICROORGANISMS8121970 33322390 PMC7764203

[B28] Logan B. (2004). Peer reviewed: Extracting hydrogen and electricity from renewable resources. *Environ. Sci. Technol.* 38 160A–167A. 10.1021/ES040468S 15180042

[B29] Lutpi N. Jahim J. Mumtaz T. Abdul P. Mohd Nor M. (2015). Physicochemical characteristics of attached biofilm on granular activated carbon for thermophilic biohydrogen production. *RSC Adv.* 5 19382–19392. 10.1039/C4RA12730G

[B30] Martínez-Fraile C. Muñoz R. Teresa Simorte M. Sanz I. García-Depraect O. (2024). Biohydrogen production by lactate-driven dark fermentation of real organic wastes derived from solid waste treatment plants. *Bioresour. Technol.* 403:130846. 10.1016/J.BIORTECH.2024.130846 38754561

[B31] Menzel T. Neubauer P. Junne S. (2023). Effect of bioaugmentation with Paenibacillus spp. and thin slurry recirculation on microbial hydrolysis of maize silage and bedding straw in a plug-flow reactor. *Biomass Conv. Biorefinery* 14 19139–19154. 10.1007/S13399-023-03958-8

[B32] Mohan S. Rao N. Prasad K. Sarma P. (2005). Bioaugmentation of an anaerobic sequencing batch biofilm reactor (AnSBBR) with immobilized sulphate reducing bacteria (SRB) for the treatment of sulphate bearing chemical wastewater. *Process. Biochem.* 40 2849–2857. 10.1016/J.PROCBIO.2004.12.027

[B33] Mohd Jamaludin N. Jamali N. Abdullah L. Idrus S. Engliman N. Abdul P. (2023). Biohydrogen production with utilisation of magnetite nanoparticles embedded in granular activated carbon from coconut shell. *Int. J. Hydrogen Energy* 48 11695–11708. 10.1016/J.IJHYDENE.2022.12.073

[B34] Montoya-Rosales J. Ontiveros-Valencia A. Esquivel-Hernández D. A. Etchebehere C. Celis L. B. Razo-Flores E. (2023). Metatranscriptomic analysis reveals the coexpression of hydrogen-producing and homoacetogenesis genes in dark fermentative reactors operated at high substrate loads. *Environ. Sci. Technol.* 57 11552–11560. 10.1021/ACS.EST.3C02066 37494704

[B35] Moreno Dávila I. Tamayo Ordoñez M. Morales Martínez T. Soria Ortiz A. Gutiérrez Rodríguez B. Rodríguez de la Garza J. A. et al. (2020). Effect of fermentation time/hydraulic retention time in a UASB reactor for hydrogen production using surface response methodology. *Int. J. Hydrogen Energy* 45 13702–13706. 10.1016/J.IJHYDENE.2019.12.137

[B36] Nikolaidis P. Poullikkas A. (2017). A comparative overview of hydrogen production processes. *Renewable Sustainable Energy Rev.* 67 597–611. 10.1016/J.RSER.2016.09.044

[B37] Noblecourt A. Christophe G. Larroche C. Fontanille P. (2018). Hydrogen production by dark fermentation from pre-fermented depackaging food wastes. *Bioresour. Technol.* 247 864–870. 10.1016/j.biortech.2017.09.199 30060424

[B38] Okonkwo O. Escudie R. Bernet N. Mangayil R. Lakaniemi A. Trably E. (2020). Bioaugmentation enhances dark fermentative hydrogen production in cultures exposed to short-term temperature fluctuations. *Appl. Microbiol. Biotechnol.* 104 439–449. 10.1007/S00253-019-10203-8 31754763 PMC6942602

[B39] Oksanen J. Simpson G. Blanchet F. Kindt R. Legendre P. Minchin P. et al. (2026). *Community Ecology Package [R package vegan version 2.7-3].* 10.32614/CRAN.PACKAGE.VEGAN

[B40] Osman A. Chen L. Yang M. Msigwa G. Farghali M. Fawzy S. et al. (2023). environmental impact, and resilience of renewable energy under a changing climate: A review. *Environ. Chem. Lett.* 21 741–764. 10.1007/s10311-022-01532-8

[B41] Park J. Anburajan P. Kumar G. Park H. Kim S. (2017). Biohydrogen production integrated with an external dynamic membrane: A novel approach. *Int. J. Hydrogen Energy* 42 27543–27549. 10.1016/J.IJHYDENE.2017.05.145

[B42] Park J. Kang H. Park K. Park H. (2018). Direct interspecies electron transfer via conductive materials: A perspective for anaerobic digestion applications. *Bioresour. Technol.* 254 300–311. 10.1016/J.BIORTECH.2018.01.095 29398288

[B43] Park J. Park J. Lee S. Yoon J. Kim S. Park H. (2020). Metabolic flux and functional potential of microbial community in an acidogenic dynamic membrane bioreactor. *Bioresour. Technol.* 305:123060. 10.1016/J.BIORTECH.2020.123060 32114306

[B44] Quintelas C. Silva B. Figueiredo H. Tavares T. (2009). Removal of organic compounds by a biofilm supported on GAC: Modelling of batch and column data. *Biodegradation* 21 379–392. 10.1007/S10532-009-9308-5 19882356

[B45] Regueira-Marcos L. Mu noz R. García-Depraect O. (2023). Continuous lactate-driven dark fermentation of restaurant food waste: Process characterization and new insights on transient feast/famine perturbations. *Bioresour. Technol.* 385:129385. 10.1016/j.biortech.2023.129385 37364653

[B46] Saady N. (2013). Homoacetogenesis during hydrogen production by mixed cultures dark fermentation: Unresolved challenge. *Int. J. Hydrogen Energy* 38 13172–13191. 10.1016/J.IJHYDENE.2013.07.122

[B47] Salmaso N. (1996). Seasonal variation in the composition and rate of change of the phytoplankton community in a deep subalpine lake (Lake Garda, Northern Italy). An application of nonmetric multidimensional scaling and cluster analysis. *Hydrobiologia* 337 49–68. 10.1007/BF00028506

[B48] Sarma S. Anand A. Dubey V. Moholkar V. (2017). Metabolic flux network analysis of hydrogen production from crude glycerol by Clostridium pasteurianum. *Bioresour. Technol.* 242 169–177. 10.1016/J.BIORTECH.2017.03.168 28456454

[B49] Seon J. Lee T. Lee S. Pham H. Woo H. Song M. (2014). Bacterial community structure in maximum volatile fatty acids production from alginate in acidogenesis. *Bioresour. Technol.* 157 22–27. 10.1016/J.BIORTECH.2014.01.072 24530946

[B50] Show K. Lee D. Chang J. (2011). Bioreactor and process design for biohydrogen production. *Bioresour. Technol.* 102 8524–8533. 10.1016/J.BIORTECH.2011.04.055 21624834

[B51] Show K. Lee D. Tay J. Lin C. Chang J. (2012). Biohydrogen production: Current perspectives and the way forward. *Int. J. Hydrogen Energy* 37 15616–15631. 10.1016/J.IJHYDENE.2012.04.109

[B52] Sim Y. Kim D. Ko J. Jung J. Kim S. (2024). Bioaugmentation with Clostridium pasteurianum for high-yield continuous bio-hydrogen production in a dynamic membrane bioreactor. *Chem. Eng. J.* 497:154709. 10.1016/J.CEJ.2024.154709

[B53] Sim Y. Yang J. Kim S. Joo H. Jung J. Kim D. et al. (2022). Effect of bioaugmentation using Clostridium butyricum on the start-up and the performance of continuous biohydrogen production. *Bioresour. Technol.* 366:128181. 10.1016/J.BIORTECH.2022.128181 36307024

[B54] Sivagurunathan P. Anburajan P. Kumar G. Kim S. (2016). Effect of hydraulic retention time (HRT) on biohydrogen production from galactose in an up-flow anaerobic sludge blanket reactor. *Int. J. Hydrogen Energy* 41 21670–21677. 10.1016/J.IJHYDENE.2016.06.047

[B55] VanFossen A. Verhaart M. Kengen S. Kelly R. (2009). Carbohydrate utilization patterns for the extremely thermophilic bacterium Caldicellulosiruptor saccharolyticus reveal broad growth substrate preferences. *Appl. Environ. Microbiol.* 75 7718–7724. 10.1128/AEM.01959-09 19820143 PMC2794124

[B56] Villanueva-Galindo E. Moreno-Andrade I. (2021). Bioaugmentation on hydrogen production from food waste. *Int. J. Hydrogen Energy* 46 25985–25994. 10.1016/J.IJHYDENE.2020.11.092

[B57] Yang J. Sim Y. Kim S. Joo H. Mašek O. Kim S. (2024). Synergetic effects of conductive materials and bacterial population in inoculum on mixed-culture biohydrogen production. *Int. J. Hydrogen Energy* 53 1293–1302. 10.1016/J.IJHYDENE.2023.12.103

[B58] Yin Y. Wang J. (2021). Predictive functional profiling of microbial communities in fermentative hydrogen production system using PICRUSt. *Int. J. Hydrogen Energy* 46 3716–3725. 10.1016/J.IJHYDENE.2020.10.246

[B59] Yu H. Mu Y. (2006). Biological hydrogen production in a UASB reactor with granules. II: Reactor performance in 3-year operation. *Biotechnol. Bioeng.* 94 988–995. 10.1002/bit.20923 16615161

[B60] Zhang J. Mao X. Yu H. Jin X. Zhang L. Du K. et al. (2025). Soil microbial community characteristics and influencing factors in Alpine Marsh Wetlands with different degradation levels in Qilian Mountain National Park, Qinghai, China. *Biology* 14:598. 10.3390/BIOLOGY14060598 40563850 PMC12189283

[B61] Zhang Z. Tay J. Show K. Yan R. Tee Liang D. Lee D. et al. (2007). Biohydrogen production in a granular activated carbon anaerobic fluidized bed reactor. *Int. J. Hydrogen Energy* 32 185–191. 10.1016/j.ijhydene.2006.08.017

[B62] Zhao B. Wang L. Li F. Hua D. Ma C. Ma Y. et al. (2010). Kinetics of d-lactic acid production by Sporolactobacillus sp. strain CASD using repeated batch fermentation. *Bioresour. Technol.* 101 6499–6505. 10.1016/J.BIORTECH.2010.03.069 20374976

[B63] Zhao B. Yue Z. Zhao Q. Mu Y. Yu H. Harada H. et al. (2008). Optimization of hydrogen production in a granule-based UASB reactor. *Int. J. Hydrogen Energy* 33 2454–2461. 10.1016/j.ijhydene.2008.03.008

[B64] Zhu L. Li W. Dong X. (2003). Species identification of genus Bifidobacterium based on partial HSP60 gene sequences and proposal of Bifidobacterium thermacidophilum subsp. porcinum subsp. nov. *Int. J. Syst. Evol. Microbiol.* 53 1619–1623. 10.1099/ijs.0.02617-0 13130059

[B65] Zinatizadeh A. Mohammadi P. Mirghorayshi M. Ibrahim S. Younesi H. Mohamed A. (2017). An anaerobic hybrid bioreactor of granular and immobilized biomass for anaerobic digestion (AD) and dark fermentation (DF) of palm oil mill effluent: Mass transfer evaluation in granular sludge and role of internal packing. *Biomass Bioenergy* 103 1–10. 10.1016/J.BIOMBIOE.2017.05.006

