# Supplementary material for: High-rate dark fermentative hydrogen production in a UASB reactor with granular activated carbon and Clostridium pasteurianum bioaugmentation
Source: Front Microbiol. 2026 Aug 4;17:1890141. doi: 10.3389/fmicb.2026.1890141 (PMC13482858; doi:10.3389/fmicb.2026.1890141)
Supplement: Supplementary file 1 [file Data_Sheet_1.docx]

**Supplementary material**

High-rate dark fermentative hydrogen production in a UASB reactor with granular activated carbon and *Clostridium* *pasteurianum* bioaugmentation

Eunjin Kim^a, 1^, Gi-Beom Kim^b, 1^,Yerim Lee^a^, Eunseo Cho^a^, Jiho Jeon^a^, Sang-Hyoun Kim^c^, Ju-Hyeong Jung^a, *^

^a^Department of Environmental Engineering, Kunsan National University, Gunsan, 54150, Republic of Korea

^b^Department of Biological and Agricultural Engineering & Texas A&M AgriLife Research Center, Texas A&M University, TX 76401, USA

^c^Department of Civil and Environmental Engineering, Yonsei University, Seoul, 03722, Republic of Korea

*Corresponding author: Tel.: +82-63-469-1873

E-mail address: [jhjung@kunsan.ac.kr](mailto:jhjung@kunsan.ac.kr)

| Reactor | Strategy | Substrate | Minimum HRT | Maximum HY  (mol H₂/mol substrate) | Maximum HPR (L/L/d) | Reference |
| --- | --- | --- | --- | --- | --- | --- |
| AFBR | GAC | Glucose  (10 g/L) | 1 h | N.R | 56.6 | [37] |
| CSTR | *C. butyricum* bioaugmentation | Glucose | 2 h | N.R | 58.57 ± 5.03 | [38] |
| **UASB** | **None (control)** | **Glucose**  **(20 g/L)** | **2 h** | **1.54±0.30** | **20.46±1.84** | **This study (UASB-C)** |
| **UASB** | **GAC + *C. pasteurianum* bioaugmentation** | **Glucose**  **(20 g/L)** | **1 h** | **1.94±0.03** | **83.08±2.34** | **This study (UASB-ACBA)** |

**Table S1.** Comparison of hydrogen production performance in continuous dark fermentation systems employing GAC, bioaugmentation, and their combined application.

**Figure S1.** Cumulative hydrogen production measured (point) and fitted by the modified Gompertz model (line) for each period.

**Figure S2.** Stress plot illustrating the relationship between observed dissimilarity and ordination distance in the NMDS analysis
